# Pharmacodynamic effects of the PARP inhibitor talazoparib (MDV3800, BMN 673) in patients with *BRCA*-mutated advanced solid tumors

**DOI:** 10.1007/s00280-023-04600-0

**Published:** 2023-11-27

**Authors:** Arjun Mittra, Geraldine H. O’ Sullivan Coyne, Jennifer Zlott, Shivaani Kummar, Robert Meehan, Lawrence Rubinstein, Lamin Juwara, Deborah Wilsker, Jiuping Ji, Brandon Miller, Tony Navas, Katherine V. Ferry-Galow, Andrea Regier Voth, Ting-Chia Chang, Shahanawaz Jiwani, Ralph E. Parchment, James H. Doroshow, Alice P. Chen

**Affiliations:** 1 Division of Cancer Treatment and Diagnosis, National Cancer Institute, National Institutes of Health, 31 Center Drive, Bethesda, MD 20892 USA; 2 Biometric Research Program, National Cancer Institute, Bethesda, MD 20892 USA; 3 Clinical Monitoring Research Program Directorate, Frederick National Laboratory for Cancer Research, Frederick, MD 21702 USA; 4 Clinical Pharmacodynamics Biomarkers Program, Applied/Developmental Research Directorate, Frederick National Laboratory for Cancer Research, Frederick, MD 21702 USA; 5 Applied/Developmental Research Directorate, Frederick National Laboratory for Cancer Research, Frederick, MD 21702 USA; 6 Molecular Characterization Laboratory, Frederick National Laboratory for Cancer Research, Frederick, MD 21702 USA; 7 Center for Cancer Research, National Cancer Institute, Bethesda, MD 20892 USA; 8 Present Address: Division of Medical Oncology, The Ohio State University, Columbus, OH 43210 USA; 9 Present Address: Division of Hematology and Medical Oncology, Knight Cancer Institute, Oregon Health & Science University, Portland, OR 97239 USA; 10 Present Address: Regeneron Pharmaceuticals, Tarrytown, NY 10591 USA

**Keywords:** Clinical trial, Targeted agent, Poly-ADP-ribosylation, DNA damage repair, Homologous recombination repair, Pharmacology

## Abstract

**Purpose:**

Talazoparib is an inhibitor of the poly (ADP-ribose) polymerase (PARP) family of enzymes and is FDA-approved for patients with (suspected) deleterious germline *BRCA1/2*-mutated, HER2‑negative, locally advanced or metastatic breast cancer. Because knowledge of the pharmacodynamic (PD) effects of talazoparib in patients has been limited to studies of PARP enzymatic activity (PARylation) in peripheral blood mononuclear cells, we developed a study to assess tumoral PD response to talazoparib treatment (NCT01989546).

**Methods:**

We administered single-agent talazoparib (1 mg/day) orally in 28-day cycles to adult patients with advanced solid tumors harboring (suspected) deleterious *BRCA1* or *BRCA2* mutations. The primary objective was to examine the PD effects of talazoparib; the secondary objective was to determine overall response rate (ORR). Tumor biopsies were mandatory at baseline and post-treatment on day 8 (optional at disease progression). Biopsies were analyzed for PARylation, DNA damage response (γH2AX), and epithelial‒mesenchymal transition.

**Results:**

Nine patients enrolled in this trial. Four of six patients (67%) evaluable for the primary PD endpoint exhibited a nuclear γH2AX response on day 8 of treatment, and five of six (83%) also exhibited strong suppression of PARylation. A transition towards a more mesenchymal phenotype was seen in 4 of 6 carcinoma patients, but this biological change did not affect γH2AX or PAR responses. The ORR was 55% with the five partial responses lasting a median of six cycles.

**Conclusion:**

Intra-tumoral DNA damage response and inhibition of PARP enzymatic activity were confirmed in patients with advanced solid tumors harboring *BRCA1/2* mutations after 8 days of talazoparib treatment.

**Supplementary Information:**

The online version contains supplementary material available at 10.1007/s00280-023-04600-0.

## Introduction

Poly-ADP-ribosylation of histones and other nuclear proteins by poly (ADP-ribose) polymerases 1 and 2 (PARP1/2) occurs after single- or double-stranded DNA damage, [1] and PARP activity is essential for the repair of single-stranded DNA breaks through the base excision repair (BER) pathway [2, 3]. The loss of BER caused by inhibitors of PARP enzymatic activity results in single-stranded DNA breaks that persist through DNA synthesis, causing replication fork collapse and single-ended DNA double-strand breaks. In *BRCA1*- or *BRCA2*- (*BRCA1/2*) deficient cells, these double-stranded breaks cannot be repaired by homologous recombination, resulting in increased chromatid breaks and cell death [4–6]. Inhibiting PARP in the setting of *BRCA1/2* loss-of-function mutations leads to synthetic lethality in *BRCA1/2*-mutated cells. An important putative mechanism for this lethality is the trapping of PARP at the site of DNA damage, which enhances the persistence of single-stranded DNA breaks that cannot then be repaired in cancer cells lacking functional homologous recombination pathways [7–9].

Several inhibitors of the PARP1/2 enzymes have entered clinical development over the last two decades. Olaparib was the first agent in this class to be approved by the FDA, and received an indication as maintenance therapy in patients with advanced ovarian cancer harboring a *BRCA* mutation who had received three or more prior lines of chemotherapy [10]. Olaparib was subsequently approved for several other indications in ovarian, breast, pancreatic, and prostate cancers [11]. Rucaparib first received approval from the FDA in 2016 as maintenance therapy for patients with advanced ovarian cancer and germline or somatic *BRCA* mutations, with a subsequent approval in *BRCA*-mutated metastatic castrate-resistant prostate cancer [12, 13]. Niraparib received FDA approval in 2017 for recurrent platinum-sensitive ovarian cancer, irrespective of *BRCA* mutation and homologous recombination deficiency status [14]. Another PARP inhibitor, veliparib, has undergone extensive clinical investigation, with 25 completed clinical trials as a single agent or in combination with chemotherapy or radiation [15]. This includes the BROCADE 3 randomized phase 3 study of the addition of veliparib or placebo to carboplatin/paclitaxel in advanced HER2-negative breast cancer patients, which demonstrated a progression free survival (PFS) benefit in the veliparib arm compared to placebo [16]. While showing promising results in this and other studies, veliparib has not yet received FDA approval.

Talazoparib (BMN 673) is a highly selective and potent PARP inhibitor that has shown robust anticancer activity in preclinical models, inhibiting PARP at a lower concentration and trapping PARP more potently and completely than the other PARP inhibitors in development, demonstrating the strongest cytotoxic effect in vitro [9, 17–22]. The phase 1, first-in-human trial of talazoparib as a single agent enrolled patients with advanced solid tumors predicted to be sensitive to PARP inhibition. These included tumors harboring germline *BRCA1/2* mutations, triple-negative breast, high-grade gynecological, castration-resistant prostate, and pancreatic cancers. The trial also included patients with Ewing sarcoma and small cell lung cancer (SCLC) based on promising preclinical data [23, 24]. A recommended phase 2 dose of 1 mg/day talazoparib was identified and thrombocytopenia, anemia, and fatigue were observed as the most common adverse events. Promising efficacy was demonstrated with overall response rates (ORR) of 50% and 42% in patients with *BRCA1/2*-mutated breast and ovarian cancers, respectively. Patients with pancreatic cancer had a modest ORR of 20%, while responses were much lower in patients with SCLC (8.7%) and Ewing sarcoma (0%). Pharmacodynamic (PD) studies assessing PARP activity in peripheral blood mononuclear cells (PBMCs) as a surrogate for intratumoral activity determined that talazoparib treatment decreased PARP enzymatic activity (PARylation) in PBMCs in a dose- and particularly exposure-dependent manner; however, this trial did not evaluate the PD effects of talazoparib within patients’ tumors [23].

The phase 2 ABRAZO trial studied single-agent talazoparib in patients with *BRCA*-mutated advanced breast cancer, divided into two cohorts. The response rate was 21% in patients who had responded to prior platinum chemotherapy and 37% in those who had received three or more platinum-free chemotherapy regimens [25]. The subsequent randomized phase 3 EMBRACA trial in patients with *BRCA1/2*-mutated advanced breast cancer showed an improvement compared to standard therapy in progression-free survival (PFS) (8.6 vs. 5.6 months) [26]. The results of this trial led the FDA to approve talazoparib for patients with deleterious or suspected deleterious germline *BRCA1/2*-mutated, HER2‑negative, locally advanced or metastatic breast cancer. There are also several ongoing phase 2 and 3 trials of talazoparib either as monotherapy or in combination with other agents, including a randomized phase 3 trial testing enzalutamide with or without talazoparib in patients with DNA damage response (DDR) gene-mutated metastatic castrate-sensitive prostate cancer (NCT04821622), and another in patients with metastatic castrate-resistant prostate cancer (NCT03395197).

The substantial data regarding the preclinical potency and clinical efficacy of talazoparib, coupled with the lack of demonstration of a primary (*i.e.*, direct) PD response to this agent in patient tumors, made talazoparib interesting to our multidisciplinary drug development group, particularly in the wake of the recognition that the agent iniparib, which was developed as a PARP inhibitor, did not inhibit PARP at clinically relevant doses and likely achieved its anti-neoplastic effects through a different mechanism of action [27, 28]. To address this knowledge gap in talazoparib pharmacology, we designed a pilot study with the primary objective of evaluating the PD effects of talazoparib in tumor using validated assays for two biomarkers in core needle biopsy specimens: intracellular levels of PARylated protein to assess target engagement and nuclear levels of γH2AX to assess DNA damage response [29]. The study also assessed the antitumor efficacy of talazoparib in patients with advanced ovarian, breast, or other solid tumors harboring (suspected) deleterious *BRCA1/2* mutations, and patients’ tumor biopsies were evaluated for E-cadherin and vimentin, phenotypic biomarkers of epithelial-mesenchymal state, to further understand the potential for carcinomas to adapt to treatment by undergoing epithelial-mesenchymal transition (EMT) and the implications of initial epithelial-mesenchymal phenotype on the response of patients to talazoparib.

### Patients and methods

#### Patient population

This single-center study enrolled adult patients with documented deleterious or suspected deleterious somatic or germline *BRCA1* or *BRCA2* mutations and histologically confirmed solid tumors. Patients with ovarian cancer were required to have received at least one prior platinum-based chemotherapeutic regimen, and those with platinum-refractory disease were not eligible. Patients with HER2-positive advanced breast cancer or ovarian cancer were required to have received at least two lines of systemic therapy in the advanced setting. All other patients must have had tumors that progressed through at least one line of standard therapy or have had no acceptable standard treatment options. Patients with metastatic disease must have received at least one line of standard treatment for metastatic disease prior to enrollment.

Additional eligibility criteria were an Eastern Cooperative Group (ECOG) performance status of 0–2, the ability to swallow whole capsules, and adequate organ and marrow function. Patients were required to have disease amenable to biopsy and be willing to undergo paired tumor biopsies. Previous treatments must have been completed at least 4 weeks prior to enrollment. Exclusion criteria included prior treatment with any PARP inhibitors, active brain metastases, or HIV-positive patients on combination antiretroviral therapy because of the potential for pharmacokinetic interactions with talazoparib.

#### Study design and treatment

This was an open-label pilot study of single-agent talazoparib with the primary objective of determining the PD effect of talazoparib treatment via evaluation of tumor biopsies and a secondary objective of determining the response rate of talazoparib treatment in patients with solid tumors harboring (suspected) deleterious *BRCA1/2* mutations. Talazoparib was supplied by the Division of Cancer Treatment and Diagnosis of the National Cancer Institute (NCI), under a cooperative research and development agreement with Medivation LLC, a Pfizer company; the drug was formerly under development with BioMarin Pharmaceutical, Inc. Talazoparib was administered orally at the FDA-approved dose of 1 mg/day daily in 28-day cycles [30]. Patients were required to maintain a diary documenting when drugs were taken but there were no restrictions on food consumption. Toxicities were graded according to the Common Terminology Criteria for Adverse Events (CTCAE version 4.0). The talazoparib dose was reduced for grade ≥ 2 non-hematologic and grade 4 hematologic toxicities. Toxicities were required to have resolved to ≤ grade 2 (except anemia, lymphopenia, or leucopenia in the absence of grade 4 neutropenia) prior to re-initiating treatment at the next lower dose level (750 or 500 µg/day). Radiologic response assessments by computerized tomography (CT) scans were performed at baseline and every 2 cycles. Tumor response was evaluated according to the Response Evaluation Criteria in Solid Tumors (RECIST version 1.1) [31]. History and physical examination and laboratory evaluations (complete blood count and serum chemistries) were performed prior to treatment, after 8 days of treatment, and at the beginning of each subsequent 28 day cycle. Investigators obtained informed consent from each participant and this trial was conducted in accordance with the Declaration of Helsinki, under an NCI-sponsored IND with institutional review board approval [ClinicalTrials.gov Identifier: NCT01989546].

#### Pharmacokinetic analyses

Plasma levels of talazoparib were measured using a validated liquid chromatography-tandem mass spectrometry assay. Metabolite levels were not assessed as talazoparib is minimally metabolized and metabolites have not been detected in plasma [32]. Blood samples were collected prior to treatment; 0.5, 1, 2, 3, 4, 6, 8, and 24 h after the first dose; 3–6 h after the day 8 dose; and before and 3–6 h after the cycle 2, day 1 dose. Standard pharmacokinetic parameters were calculated using a noncompartmental method (Phoenix WinNonlin^®^ 6.3; Certera, Princeton, NJ).

#### Pharmacodynamic endpoint analyses

Mandatory paired tumor biopsies (18-gauge core needle biopsies, 2 cores) were obtained from subjects at baseline and 3 to 6 h after treatment on day 8 of cycle 1. In addition, optional tumor biopsies were collected at the time of disease progression. All biopsy cores were flash frozen within 2 min of collection. One core from each time point was extracted and analyzed for levels of PAR attached to macromolecules (a PARP reaction product) using a previously validated, fit-for-purpose ELISA method [33, 34]. The second core was thawed and fixed in neutral buffered formalin, paraffin-embedded (FFPE), sectioned, and Hematoxylin and Eosin (H&E) stained for pathologist review. Biopsies with sufficient viable tumor content (> 5%) were analyzed for the DNA damage marker γH2AX, as described previously [29, 35]. The EMT markers E-cadherin and vimentin were also assessed using a fit-for-purpose immunofluorescence assay (IFA), validation of which has been described by Navas, et al. [36]. Briefly, flanking 5 µM serial sections were stained either with H&E for pathologist review to define regions of interest with viable tumor content or with monoclonal antibody-fluorescent dye conjugates of E-cadherin, vimentin, and β-catenin, along with 40,6-diamidino-2-phenylindole (DAPI). After imaging, a custom image analysis algorithm was used to create a detailed tumor-stroma segmentation map of each image by combining β-catenin staining intensity with cellular morphology. Then, E-cadherin and vimentin staining for individual, pathologist-defined regions of interest, areas of marker overlap, and total number of nuclei (*i.e.*, cells) were reported for each biopsy and patient. Mann–Whitney statistical tests were performed using GraphPad Prism 9.

Whole blood was collected for isolation and analysis of circulating tumor cells (CTCs) before treatment, twice after the day 1 treatment, once after the day 8 treatment, and once after the first treatment of cycle 2. Later patients also had blood collected prior to the first treatment of every subsequent cycle and at disease progression. Blood samples were enriched for CTCs using the ApoStream System (ApoCell) and enriched cell fractions were analyzed using slide-based multiplex immunofluorescence assays with appropriate tumor and phenotypic markers based on the patient’s diagnosis.

#### Statistical analysis

The trial design planned to accrue up to 24 patients to assure at least 12 patients with matched, evaluable baseline and day 8 biopsies. The primary endpoint was the percent of patients who achieve a significant PD response, defined as at least 4% tumor nuclear area positive (NAP) for γH2AX in the day 8 biopsy [29]. In previous clinical testing, a γH2AX NAP value ≥ 4% has been seen in tumor tissues from untreated patients in less than 5% of cases. Observing at least 3 out of the 16 (19%) patients with evaluable baseline and day 8 biopsies achieving this level of PD response would declare the agent promising by means of the PD assay. This design gives 90% power to detect a true 30% PD response rate, across patients with less than 0.04 probability of a false positive in the event that the agent has no effect and the true likelihood of such a PD response, for an individual patient, is less than 5%.

## Results

### Patient population and disposition

**Fig. 1:**
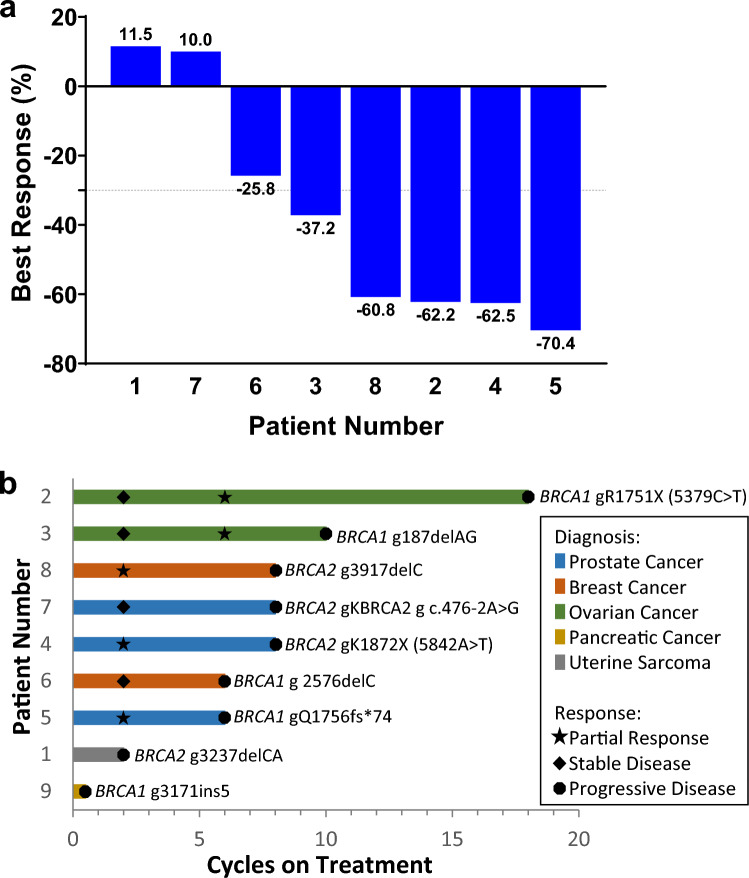
Patient outcomes. **a** Best proportion change in RECIST measurements from baseline (listed below each bar) is plotted for all patients except for patient #9, who left the trial due to clinical progression before their first restaging. **b** Number of cycles on treatment for all patients colored by diagnosis. Response and *BRCA1/2* eligibility mutation are shown for each patient

A total of nine patients, each with a unique deleterious or suspected deleterious somatic *BRCA1/2* mutation (Supplemental Figure S1), were enrolled and treated when, due to a change in pharmaceutical sponsorship and the signs of clinical and PD activities already seen, the decision was made to terminate the trial. All patients had metastatic tumors: three were diagnosed with prostate cancer, two with ovarian cancer, two with breast cancer, one with uterine sarcoma, and one with pancreatic cancer (Table 1). Most patients had been heavily pre-treated with a median of 5 prior lines of systemic therapy (range: 1–9). One patient (patient #9, pancreatic cancer) had clinical progression and was taken off study during the first treatment cycle, after their day 8 biopsy was collected. The remaining 8 patients eventually came off study due to progressive disease. Mean time on study was seven cycles (range: 0–18), and five of the nine (55%) had documented partial responses (PR) lasting between 4 and 12 cycles (median: 6 cycles). A further two patients had a best response of stable disease (SD) for 4 to 6 cycles, while the remaining two patients progressed, respectively, during the first cycle and at the first scheduled restaging (2 cycles) (Fig. 1).

**Table 1:** Patient demographics

Patient	Sex	Age	Diagnosis	Eligibility Mutation	Prior Lines of Therapy	Prior Platinum
1	F	69	Uterine Sarcoma	BRCA2 g3237delCA	5	Y
2	F	59	Ovarian	BRCA1 gR1751X (5379C > T)	9	Y
3	F	65	Ovarian	BRCA1 g187delAG	8	Y
4	M	73	Prostate	BRCA2 gK1872X (5842A > T)	1	N
5	M	61	Prostate	BRCA1 g Q1756fs*74	6	N
6	F	52	Breast	BRCA1 g2576delC	7	Y
7	M	61	Prostate	BRCA2 g c.476-2A > G	1	N
8	F	33	Breast	BRCA2 g3917delC	4	N
9	M	73	Pancreatic	BRCA1 g3171ins5	1	Y

### Adverse events

Treatment-related adverse events (trAE) are described in Table 2. The most common grade 2 or higher trAEs were related to decreased blood counts: white blood cell count decrease (67%), neutrophil count decrease (67%), lymphocyte count decrease (56%), platelet count decrease (44%), and anemia (33%). Grade 3 or 4 trAEs also primarily involved blood counts (lymphocyte decrease, platelet count decrease, neutrophil count decrease, and anemia), while 1 patient experienced an increase in alkaline phosphatase. There were no grade 5 events, and no patients were taken off study for toxicity.

**Table 2:** Adverse events

Adverse event*	Grade 2	Grade 3	Grade 4	Total
Alkaline phosphatase increased	–	1	–	1 (11%)
Anemia	–	2	1	3 (33%)
Anorexia	1	–	–	1 (11%)
Fatigue	2	–	–	2 (22%)
Fever	1	–	–	1 (11%)
Lymphocyte count decreased	4	1	–	5 (56%)
Nausea	2	–	–	2 (22%)
Neutrophil count decreased	4	2	–	6 (67%)
Platelet count decreased	3	–	1	4 (44%)
Tinnitus	1	–	–	1 (11%)
Weight loss	1	–	–	1 (11%)
White blood cell decreased	5	1	–	6 (67%)

*Worst grade (≥ grade 2) per patient that was at least possibly related to study agent is reported

### Pharmacokinetic analysis

**Fig. 2:**
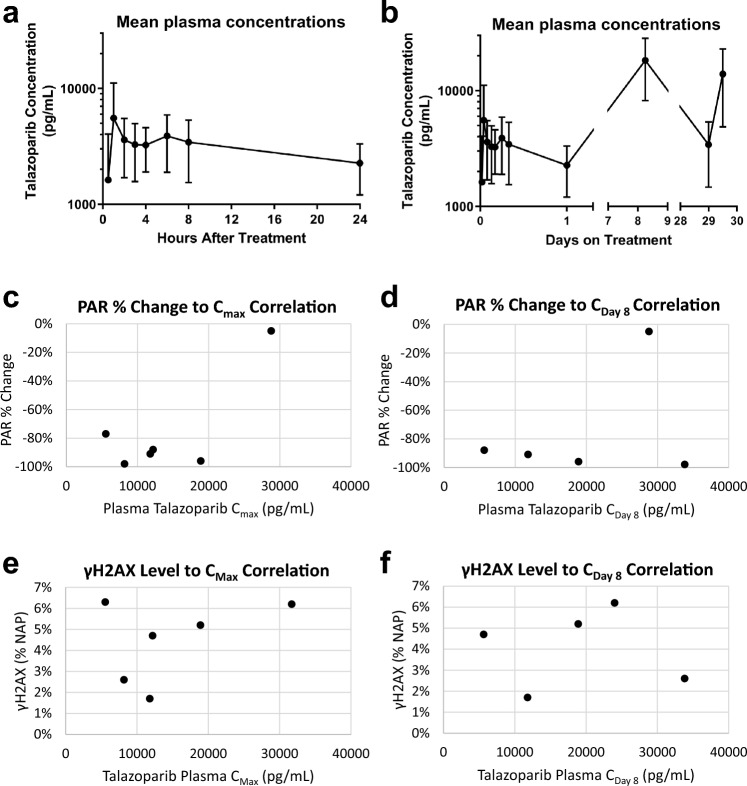
Pharmacokinetic analysis. Average talazoparib plasma levels (± standard deviation) for all 9 patients **a** during the 24 h following administration of the first dose and **b** during the entire collection period. Blood samples were collected prior to treatment; 0.5, 1, 2, 3, 4, 6, 8, and 24 h after the first dose; 3 to 6 h after the day 8 dose; and before and 3 to 6 h after the cycle 2, day 1 dose. **c** The most significant correlation between individual patient plasma exposure and PD effect was observed with the talazoparib maximal concentration (C_Max_) and the proportion of PAR inhibition observed at day 8 compared to baseline (0.76 correlation [0.25, 0.94 80% CI]). **d** No correlation between day 8 plasma talazoparib levels (C_Day 8_) and PAR inhibition was observed (0.35 correlation [− 0.5, 0.85 80% CI]), and neither did **e** C_Max_ nor **f** C_Day 8_ talazoparib levels correlate with individual patient γH2AX levels (− 0.03 correlation [− 0.73, 0.7 80% CI] and 0.39 correlation [− 0.32, 0.82 80% CI], respectively)

As no large-scale studies of human talazoparib pharmacokinetics had yet been published at the time this study was designed, plasma levels of talazoparib were measured with the hope of exploring the exposure–response relationships for talazoparib and its active metabolites with measures of effectiveness, toxicity, and PD biomarkers. The mean talazoparib plasma concentration–time profiles of once-daily talazoparib are shown in Fig. 2a and b. Considerable interpatient variability in talazoparib plasma concentrations was documented but the only correlation observed between plasma exposure and PD response in individual patient tumors was seen between the proportion of PAR inhibition to the talazoparib maximal concentration (0.76 correlation [0.25, 0.94 80% CI]; Fig. 2c–f). Such correlations are difficult to observe as they rely on all patients achieving maximal PD effect at the same time after treatment and collection of the biopsy at that timepoint. The observed talazoparib PK profiles are consistent with recently published results; [37] however, the small number of patients precludes drawing any meaningful conclusions from these data.

### Pharmacodynamic analysis

Because the primary objective of the trial was the PD response of the tumor to talazoparib treatment, tumor biopsies were mandatory at baseline and on day 8. Tissue cores suitable for γH2AX assessment were obtained from 6 of 9 (67%) baseline biopsies collected and 6 of 8 (75%) day 8 biopsies collected, with exclusions due to very low (< 5%) viable tumor cell content or significant tissue damage (Supplemental Table S1). One patient (#8) underwent biopsy procedures only at baseline and disease progression (approximately 24 h after the last dose of talazoparib) and was therefore excluded from the primary PD biomarker analysis. Paired biopsy cores suitable for analysis of PARylation levels were obtained in 7 cases (78%), including the baseline/progression biopsy pair from patient #8.

### Tumor γH2AX response

**Fig. 3:**
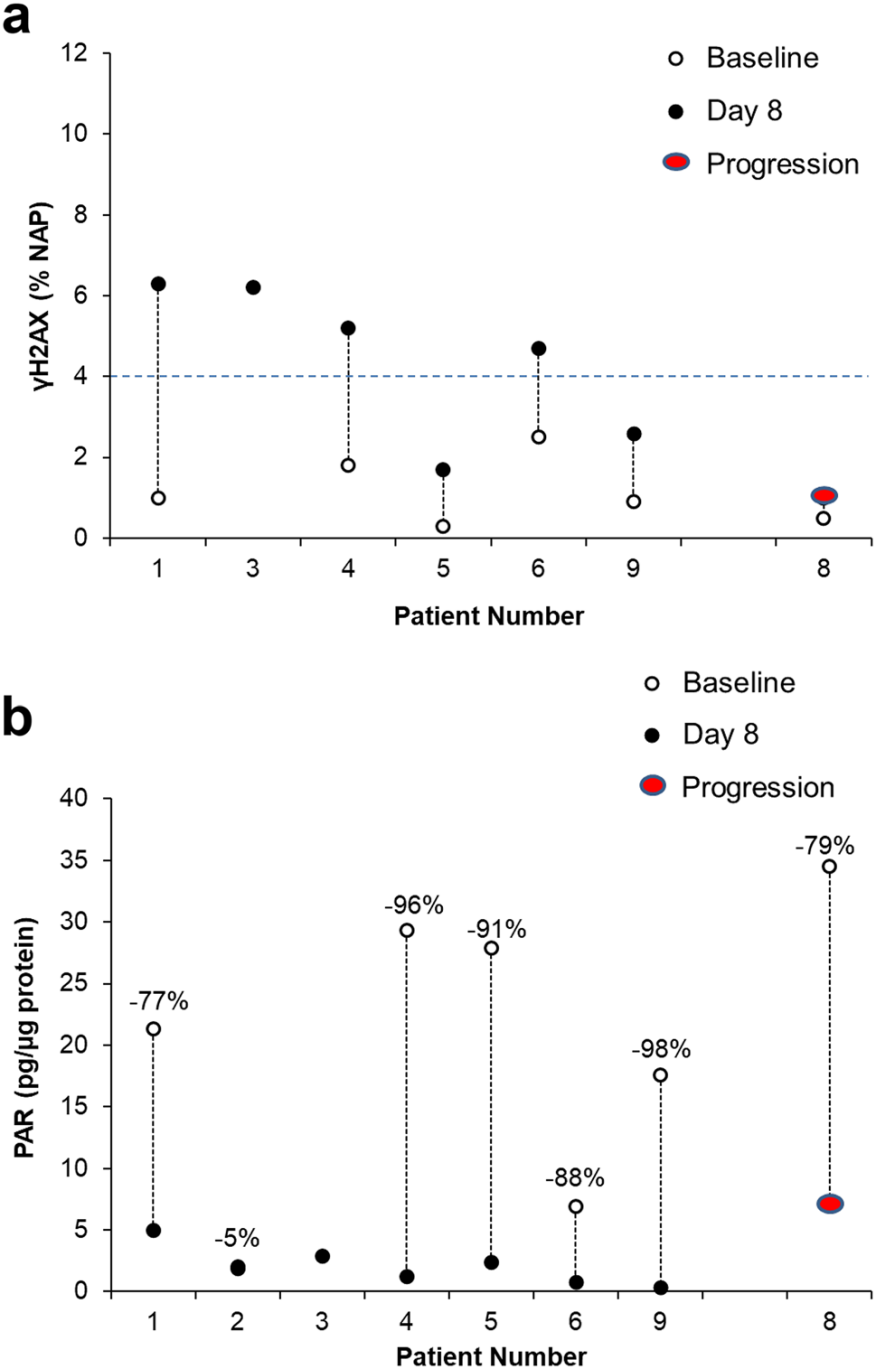
Tumoral γH2AX and PAR changes. **a** Nuclear γH2AX levels (the proportion of nuclear area positive, or %NAP, for γH2AX) and **b** PAR levels in evaluable tumor biopsies collected at baseline and 3 to 6 h after talazoparib treatment on day 8, except for patient #8, whose on-treatment biopsy was taken at progression (after cycle 8, 24 h after last dose). The blue dotted line in (**a**) represents the γH2AX effect level threshold established in Wilsker et al.[29] The proportion change in PAR from before to after treatment is shown above each patient’s data points in (**b**). More tumor biopsy cores were evaluable with the validated PAR assay than the γH2AX assay because some biopsy cores for the γH2AX immunofluorescence assay were judged to have insufficient viable tumor cellularity for image analysis during the required pathologist’s review

The study’s primary endpoint was identifying the proportion of patients achieving significant tumoral response of the nuclear PD biomarker of double-stranded DNA damage γH2AX (≥ 4.0% NAP) after 8 days of treatment. Although baseline γH2AX in the biopsy from patient #3 was not evaluable due to inadequate viable tumor cell content, baseline γH2AX in the 6 evaluable cases was < 4.0% (range, 0.3–2.5% NAP), consistent with previous case series that were not selected for *BRCA1/2* defects [29]. Talazoparib treatment resulted in elevated levels of nuclear γH2AX in 4 of the 6 evaluable cases (67%), exceeding the study design threshold of 19% (1-sided significance level of *p* = 0.014) and indicating drug-induced DNA damage and the presence of double-strand breaks (Fig. 3a and Supplemental Table S1). The only patient (patient #8) with a biopsy at disease progression did not exhibit a γH2AX response, potentially due to the time elapsed since the final talazoparib treatment (approximately 24 h) or some type of acquired talazoparib resistance.

### Tumor PARylation response

The primary pharmacodynamic effect (*i.e.*, the direct consequence of the inhibition of PARP1/2 enzymes) of talazoparib was assessed by measuring PARylation levels within the tumor. PAR levels decreased by 77–98% in 5 of 6 patients with evaluable paired biopsies, indicating that intra-tumoral talazoparib levels were sufficient 3 to 6 h after talazoparib treatment on day 8 to significantly inhibit PARP enzymatic activity (Fig. 3b). Similar PARylation responses were observed regardless of clinical or nuclear γH2AX response (Supplemental Table S1). PARylated product in patient #8 at progression (24 h after their last dose of talazoparib) was decreased by 79%, suggesting a sustained intra-tumoral effect consistent with drug PK, yet no evidence of DNA double-strand breaks was observed in the second biopsy core evaluated for γH2AX. Patient #2, who exhibited the most durable PR (12 cycles), was the only case in which significant PARylation inhibition was not observed; however, this may be due to their particularly low baseline PARylation levels. Unfortunately, this patients’ second-pass biopsies evaluated for γH2AX did not contain sufficient viable tumor cell content for valid analysis, so we were not able to explore the potential connection between low baseline PARylation and talazoparib treatment-induced DNA damage (Supplemental Table S1).

### Epithelial-mesenchymal phenotype in tumor and CTCs

**Fig. 4:**
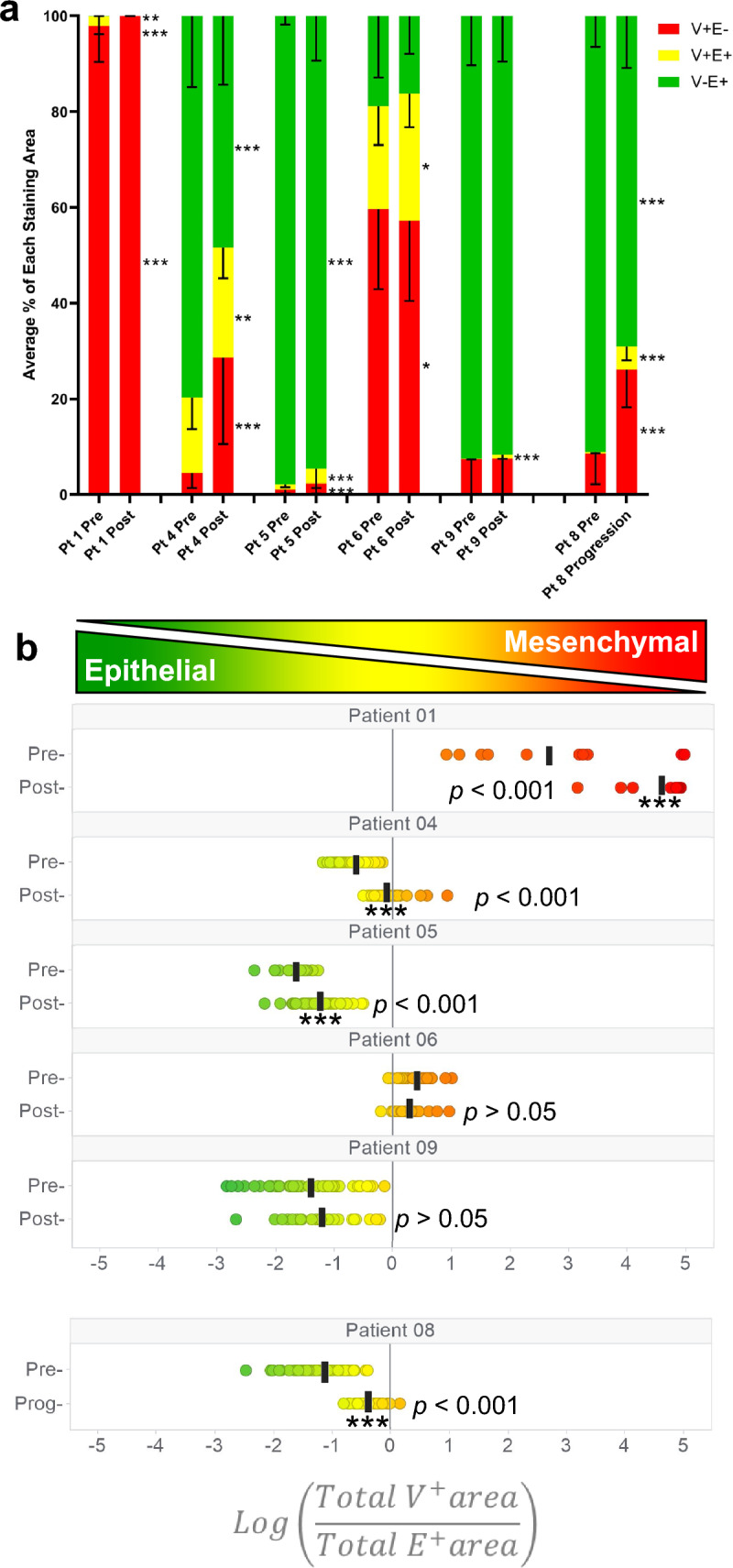
Tumor epithelial-mesenchymal phenotypes. EMT-IFA results from tumor biopsies collected at baseline and on-treatment in patients that had suitable paired biopsy samples with sufficient viable tumor cellularity for analysis: **a** Proportion of tumor area staining positive for vimentin only (V + E −, red), E-cadherin only (V − E + , green), co-localized vimentin and E-cadherin (V + E + , yellow); **b** The log(V/E) ratio of vimentin and E-cadherin-stained areas within regions of interest (ROIs) in a tissue section. In each case, significance, as calculated by the Mann–Whitney test treating each ROI as a separate observation, is shown with asterisks (* *p* < 0.05; ** *p* < 0.01; *** *p* < 0.001). The on-treatment biopsy for patient #8 was taken at progression (after cycle 8, 24 h after last dose), not day 8 as for all other patients, and therefore is separately analyzed

Significant shifts towards a more mesenchymal phenotype (as indicated by an increase in the proportion of the mesenchymal marker vimentin to epithelial marker E-cadherin [36]) after talazoparib treatment were observed in the paired biopsies of 2 of the 4 carcinoma patients evaluable for the primary PD endpoint, as well as in the progression biopsy of patient #8 (Fig. 4). As expected, the uterine sarcoma of patient #1 was entirely mesenchymal before and after treatment. While statistically significant, these biological changes were relatively modest; there were no cases of carcinoma tumors converting completely from an epithelial to a mesenchymal phenotype. This could be because day 8 is early to observe a complete phenotypic change, but the similar magnitude of the change seen at progression in patient #8 argues against there being an overall phenotypic conversion at any point during talazoparib treatment. Furthermore, the ranges of nuclear γH2AX response and PARylation inhibition were similar between carcinomas that did and did not display epithelial-mesenchymal shifts (Supplemental Table S1).

**Fig. 5:**
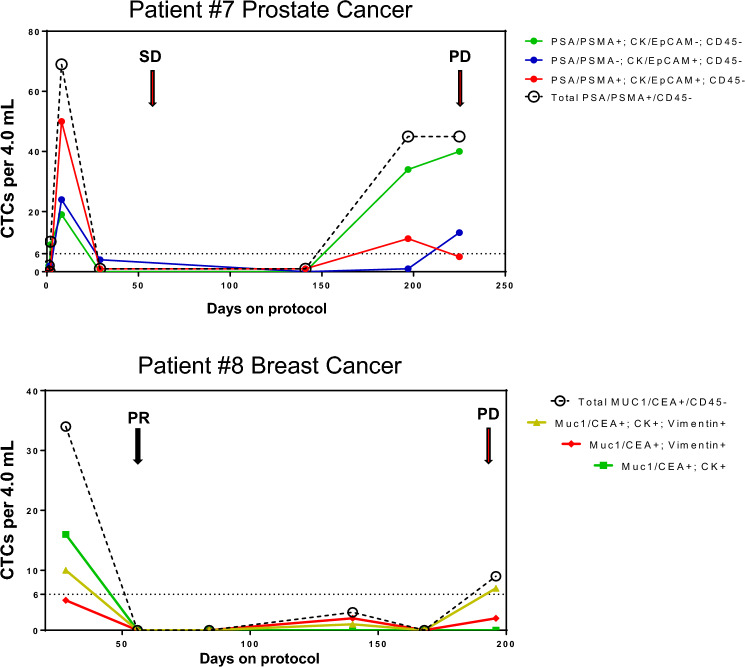
Longitudinal CTC collections. Venous blood specimens collected from patients #7 and #8 were analyzed for CTC numbers and histology- and EMT-related phenotypic markers throughout the course of treatment and progression. The dotted line represents the lower limit of quantitation (LLQ), which CTC counts at most timepoints were below for both patients; however, CTC counts rose at (patient #8) or near (patient #7) the time of disease progression and the distribution of epithelial-mesenchymal-transitional cell phenotypes differ between enrollment and progression

Two patients underwent longitudinal analysis of subpopulations of circulating tumor cells (CTC) in peripheral blood (1 SD and 1 PR). In both cases, early CTC numbers were relatively high, then low/not measurable while the drug was active, and rose in the last 1–2 cycles before progression (Fig. 5). In the case of patient #8 where epithelial-mesenchymal phenotype was evaluable both in tumor biopsy and CTCs, the trend towards a more mesenchymal phenotype at progression was echoed in the CTC analysis as the mesenchymal (Muc1/CEA^+^, vimentin^+^, CK^−^) and transitional (Muc1/CEA^+^, vimentin^+^, CK^+^) subpopulations of breast cancer CTCs became the dominant phenotypes at progression over the epithelial subpopulation of CTCs (Muc1/CEA^+^, vimentin^−^, CK^+^) that was the dominant phenotype at time of enrollment. Although a biopsy specimen is not available to corroborate the CTC finding, patient #7 showed a similar pattern, with epithelial phenotype dominating at enrollment but the mesenchymal phenotype becoming dominant by progression. Thus, 4 of 6 carcinoma patients showed evidence of talazoparib-induced EMT via either tumor biopsy or CTCs.

## Discussion

Clinical trials have shown the activity of the PARP inhibitor talazoparib in patients with *BRCA*-mutated cancers; [23, 26, 38] however, neither the inhibition of PAR nor the predicted damage to DNA has previously been demonstrated within patient tumor tissues. Our data show that single-agent talazoparib leads to a strong suppression (77–96% decrease) of tumoral PARylation at day 8 of treatment in a heavily pre-treated population with advanced disease harboring *BRCA1/2* mutations. Furthermore, a biopsy obtained at the time of tumor progression (patient #8) also had markedly suppressed levels of PAR, suggesting that this effect is sustained and was likely not altered when resistance to talazoparib developed in this patient. Interestingly, the only patient whose tumor PARylation was not significantly inhibited after talazoparib treatment (Patient #2) also exhibited a very low baseline level of PARylated product. This was lower than the drug-suppressed tumor levels of PARylated product on study day 8 in 4 of 6 molecular responders (including Patient #8 sampled at progression). Given the durable PR observed for this patient, it is possible that Patient #2’s disease evolved toward a PARP1/2-independent mechanism of survival or that their particular *BRCA1* mutation does not affect homologous recombination (although this truncated gene product is designated as pathogenic in the ClinVar database) [39]. Alternatively, it is possible that talazoparib quickly killed these cancer cells with naturally insufficient PARP1/2 activity by study day 8, leaving non-viable tumor tissue at the time of the on-treatment biopsy.

In addition to PARylation effects, talazoparib treatment also increases nuclear γH2AX foci, a biomarker of double-strand DNA damage [40]. γH2AX belongs to the H2A family of histones and has been validated as a marker of DNA damage, especially after treatment with genotoxic agents. Damage to DNA or alteration of DNA metabolism leads to the formation of DNA double-strand breaks, which in turn cause rapid phosphorylation of γH2AX and the formation of a focus at the site of the DNA double-strand break. Thus, measurement of γH2AX levels can be used to identify the extent of DNA damage [41]. Our data show that levels of intra-tumoral γH2AX were increased by day 8 of treatment, well before a detectable tumor response, suggesting rapid and increased DNA double-stranded breaks as a mechanism of tumor cell cytotoxicity.

In vitro, talazoparib produces PARP-mediated cytotoxicity more potently and extensively than either olaparib or rucaparib while also demonstrating superior trapping of PARP at the site of DNA damage [9, 17–21]. This suggests that the inhibition of intracellular PAR may not be the only contributor to tumor cytotoxicity, or even the most important; however, directly assessing PARP trapping in patient specimens using laboratory methods developed for in vitro experiments with tumor cell lines has proven difficult. Researchers continue to search for a suitable secondary biomarker of PARP trapping that can be used in patient tissue samples, but no consensus has yet been reached [42]. Our study was designed before the importance of PARP trapping became clear (or clinical assays to measure it existed) and was not designed to answer questions about this mechanism. Instead, our group is currently probing this question with an ongoing, PD-driven clinical trial (NCT04550494) assessing Rad51 activation, a biomarker best known for its part in homologous recombination but whose role in replication fork protection may prove integral after PARP trapping [42].

Consistent with this mechanistic evidence of target engagement and its downstream consequences on DNA integrity, once daily talazoparib dosing demonstrated clinical activity in patients with different cancer histologies and *BRCA1/2* mutations, including those who had received prior platinum chemotherapy. Talazoparib was generally well tolerated with no grade 5 events and no patients needing to be taken off trial for adverse events. In keeping with prior clinical trial results, the most common trAEs were related to decreased blood cell counts. Five patients experienced PRs, resulting in an ORR of 55%. Another two patients had a best response of stable disease for 4 to 6 cycles. Our data align with the ORR of 62.5% in breast cancer patients with *BRCA1/2* mutations treated with talazoparib in the phase 3 EMBRACA trial [26]. Pharmacokinetic characteristics were also similar to those of prior clinical trials [23].

Epithelial–mesenchymal transition (EMT) plays a vital role in the malignant process and progression of many carcinomas, and biomarkers of EMT are associated with poor patient prognosis in a number of carcinoma types [43]. EMT and the epithelial-mesenchymal phenotype of carcinoma cells more broadly may also be of particular importance in understanding acquired drug resistance. The expression of mesenchymal-like features resulting from complete or partial EMT can cause increased resistance to chemotherapeutic and targeted agents, therefore driving acquired resistance such as in the case of PARP inhibitor resistance in models of *BRCA2*-mutated mammary tumors [44–46]. Additionally, the capability of cancer stem cells to seed and regenerate tumors may also be attributable to their primarily mesenchymal phenotype, itself a product of EMT [43, 47, 48]. In the present clinical study, we observed a statistically significant but biologically modest drug-induced transition of carcinomas toward a mesenchymal phenotype in two of four cases at day 8 and two of two cases at progression (one of these based only on CTC analysis). The EMT detected on day 8 did not appear to influence patients’ clinical or molecular (PARylation or γH2AX) responses to talazoparib. PARP enzymes have been linked to the EMT program in several ways, including PARP1 ADP-ribosylation of Sma-mothers against decapentaplegic (Smad) family transcription factors dissociating Smad complexes from gene regulatory regions [49]. Because Smad proteins mediate broadly pro-EMT transcriptional program, [50] PARP1 activity would generally suppress EMT and inhibition of PARP enzymatic activity could create a more permissive environment for EMT to occur. Whether such a mechanism is behind the observed movement towards mesenchymal phenotypes in carcinoma tumor tissues after talazoparib treatment or whether the drug preferential kills more sensitive epithelial tumor cells and thereby selects for more inherently resistant vimentin-positive mesenchymal phenotype cells unfortunately cannot be determined by the assay used this study. It may also be important to determine whether talazoparib exhibits the same potency and efficacy regardless of a tumor’s initial epithelial–mesenchymal phenotype, but this would require a larger sample size to investigate.

A limitation of this study was the inability to meet the initially planned accrual goal after the new pharmaceutical company sponsor of the agent discontinued support. Additionally, biopsy pairs suitable for PD evaluations were not available in 3 of the 9 enrolled patients. Despite these limitations, the trial met its predefined primary endpoint of 3 or more patients displaying a significant tumoral PD response. The observations of early and robust suppression of intra-tumoral PARylated macromolecules and elevated nuclear γH2AX levels after single-agent talazoparib in *BRCA1/2*-mutated tumors provide the first insights into the clinical mechanism of action of this approved drug, which underlies its clinical efficacy.

### Supplementary Information

Below is the link to the electronic supplementary material.

Supplementary file1 (DOCX 1214 KB)

## Data Availability

The data generated in this study are included in this published article (and its supplementary information files) and are publicly available in the U.S. National Library of Medicine’s ClinicalTrials.gov database at https://clinicaltrials.gov/ct2/show/results/NCT01989546.

## References

[1] O'Sullivan CC, Moon DH, Kohn EC, Lee JM (2014). Beyond breast and ovarian cancers: PARP inhibitors for BRCA mutation-associated and BRCA-like solid tumors. Front Oncol.

[2] Schreiber V, Ame JC, Dolle P, Schultz I, Rinaldi B, Fraulob V, Menissier-de Murcia J, de Murcia G (2002). Poly(ADP-ribose) polymerase-2 (PARP-2) is required for efficient base excision DNA repair in association with PARP-1 and XRCC1. J Biol Chem.

[3] Memisoglu A, Samson L (2000). Base excision repair in yeast and mammals. Mutat Res.

[4] Bryant HE, Schultz N, Thomas HD, Parker KM, Flower D, Lopez E, Kyle S, Meuth M, Curtin NJ, Helleday T (2005). Specific killing of BRCA2-deficient tumours with inhibitors of poly(ADP-ribose) polymerase. Nature.

[5] Farmer H, McCabe N, Lord CJ, Tutt AN, Johnson DA, Richardson TB, Santarosa M, Dillon KJ, Hickson I, Knights C, Martin NM, Jackson SP, Smith GC, Ashworth A (2005). Targeting the DNA repair defect in BRCA mutant cells as a therapeutic strategy. Nature.

[6] Iglehart JD, Silver DP (2009). Synthetic lethality–a new direction in cancer-drug development. N Engl J Med.

[7] Lord CJ, Ashworth A (2017). PARP inhibitors: synthetic lethality in the clinic. Science.

[8] Helleday T (2011). The underlying mechanism for the PARP and BRCA synthetic lethality: clearing up the misunderstandings. Mol Oncol.

[9] Murai J, Huang SY, Das BB, Renaud A, Zhang Y, Doroshow JH, Ji J, Takeda S, Pommier Y (2012). Trapping of PARP1 and PARP2 by clinical PARP inhibitors. Cancer Res.

[10] Kim G, Ison G, McKee AE, Zhang H, Tang S, Gwise T, Sridhara R, Lee E, Tzou A, Philip R, Chiu HJ, Ricks TK, Palmby T, Russell AM, Ladouceur G, Pfuma E, Li H, Zhao L, Liu Q, Venugopal R, Ibrahim A, Pazdur R (2015). FDA approval summary: olaparib monotherapy in patients with deleterious germline BRCA-mutated advanced ovarian cancer treated with three or more lines of chemotherapy. Clin Cancer Res.

[11] Slade D (2020). PARP and PARG inhibitors in cancer treatment. Genes Dev.

[12] Balasubramaniam S, Beaver JA, Horton S, Fernandes LL, Tang S, Horne HN, Liu J, Liu C, Schrieber SJ, Yu J, Song P, Pierce W, Robertson KJ, Palmby TR, Chiu HJ, Lee EY, Philip R, Schuck R, Charlab R, Banerjee A, Chen XH, Wang X, Goldberg KB, Sridhara R, Kim G, Pazdur R (2017). FDA approval summary: rucaparib for the treatment of patients with deleterious BRCA mutation-associated advanced ovarian cancer. Clin Cancer Res.

[13] Anscher MS, Chang E, Gao X, Gong Y, Weinstock C, Bloomquist E, Adeniyi O, Charlab R, Zimmerman S, Serlemitsos-Day M, Ning YM, Mayrosh R, Fuller B, Trentacosti AM, Gallagher P, Bijwaard K, Philip R, Ghosh S, Fahnbulleh F, Diggs F, Arora S, Goldberg KB, Tang S, Amiri-Kordestani L, Pazdur R, Ibrahim A, Beaver JA (2021). FDA approval summary: rucaparib for the treatment of patients with deleterious BRCA-Mutated metastatic castrate-resistant prostate cancer. Oncologist.

[14] Sisay M, Edessa D (2017). PARP inhibitors as potential therapeutic agents for various cancers: focus on niraparib and its first global approval for maintenance therapy of gynecologic cancers. Gynecol Oncol Res Pract.

[15] George RR, Thomas R, Davice A, Mathew MS (2022). Veliparib for the treatment of solid malignancies. J Oncol Pharm Pract.

[16] Diéras V, Han HS, Kaufman B, Wildiers H, Friedlander M, Ayoub JP, Puhalla SL, Bondarenko I, Campone M, Jakobsen EH, Jalving M, Oprean C, Palácová M, Park YH, Shparyk Y, Yañez E, Khandelwal N, Kundu MG, Dudley M, Ratajczak CK, Maag D, Arun BK (2020). Veliparib with carboplatin and paclitaxel in BRCA-mutated advanced breast cancer (BROCADE3): a randomised, double-blind, placebo-controlled, phase 3 trial. Lancet Oncol.

[17] Shen Y, Rehman FL, Feng Y, Boshuizen J, Bajrami I, Elliott R, Wang B, Lord CJ, Post LE, Ashworth A (2013). BMN 673, a novel and highly potent PARP1/2 inhibitor for the treatment of human cancers with DNA repair deficiency. Clin Cancer Res.

[18] Andrei AZ, Hall A, Smith AL, Bascunana C, Malina A, Connor A, Altinel-Omeroglu G, Huang S, Pelletier J, Huntsman D, Gallinger S, Omeroglu A, Metrakos P, Zogopoulos G (2015). Increased in vitro and in vivo sensitivity of BRCA2-associated pancreatic cancer to the poly(ADP-ribose) polymerase-1/2 inhibitor BMN 673. Cancer Lett.

[19] Herriott A, Tudhope SJ, Junge G, Rodrigues N, Patterson MJ, Woodhouse L, Lunec J, Hunter JE, Mulligan EA, Cole M, Allinson LM, Wallis JP, Marshall S, Wang E, Curtin NJ, Willmore E (2015). PARP1 expression, activity and ex vivo sensitivity to the PARP inhibitor, talazoparib (BMN 673), in chronic lymphocytic leukaemia. Oncotarget.

[20] Wang B, Chu D, Feng Y, Shen Y, Aoyagi-Scharber M, Post LE (2016). Discovery and Characterization of (8S,9R)-5-Fluoro-8-(4-fluorophenyl)-9-(1-methyl-1H-1,2,4-triazol-5-yl)-2,7,8,9-te trahydro-3H-pyrido[4,3,2-de]phthalazin-3-one (BMN 673, Talazoparib), a Novel, Highly Potent, and Orally Efficacious Poly(ADP-ribose) Polymerase-1/2 Inhibitor, as an Anticancer Agent. J Med Chem.

[21] Murai J, Huang SY, Renaud A, Zhang Y, Ji J, Takeda S, Morris J, Teicher B, Doroshow JH, Pommier Y (2014). Stereospecific PARP trapping by BMN 673 and comparison with olaparib and rucaparib. Mol Cancer Ther.

[22] Mittra A, Doroshow JH, Chen AP (2021) BRCA Mutation and PARP Inhibitors. In: Handbook of Therapeutic Biomarkers in Cancer. Jenny Stanford Publishing, pp 533–575

[23] de Bono J, Ramanathan RK, Mina L, Chugh R, Glaspy J, Rafii S, Kaye S, Sachdev J, Heymach J, Smith DC, Henshaw JW, Herriott A, Patterson M, Curtin NJ, Byers LA, Wainberg ZA (2017). Phase I, dose-escalation, two-part trial of the PARP inhibitor talazoparib in patients with advanced germline BRCA1/2 mutations and selected sporadic cancers. Cancer Discov.

[24] Hobbs EA, Litton JK, Yap TA (2021). Development of the PARP inhibitor talazoparib for the treatment of advanced BRCA1 and BRCA2 mutated breast cancer. Expert Opin Pharmacother.

[25] Turner NC, Telli ML, Rugo HS, Mailliez A, Ettl J, Grischke EM, Mina LA, Balmana J, Fasching PA, Hurvitz SA, Wardley AM, Chappey C, Hannah AL, Robson ME, Group AS (2019). A phase II study of talazoparib after platinum or cytotoxic nonplatinum regimens in patients with advanced breast cancer and germline BRCA1/2 mutations (ABRAZO). Clin Cancer Res.

[26] Litton JK, Rugo HS, Ettl J, Hurvitz SA, Goncalves A, Lee KH, Fehrenbacher L, Yerushalmi R, Mina LA, Martin M, Roche H, Im YH, Quek RGW, Markova D, Tudor IC, Hannah AL, Eiermann W, Blum JL (2018). Talazoparib in patients with advanced breast cancer and a germline BRCA mutation. N Engl J Med.

[27] Patel AG, De Lorenzo SB, Flatten KS, Poirier GG, Kaufmann SH (2012). Failure of iniparib to inhibit poly(ADP-Ribose) polymerase in vitro. Clin Cancer Res.

[28] Mateo J, Ong M, Tan DS, Gonzalez MA, de Bono JS (2013). Appraising iniparib, the PARP inhibitor that never was–what must we learn?. Nat Rev Clin Oncol.

[29] Wilsker DF, Barrett AM, Dull AB, Lawrence SM, Hollingshead MG, Chen A, Kummar S, Parchment RE, Doroshow JH, Kinders RJ (2019). Evaluation of pharmacodynamic responses to cancer therapeutic agents using DNA damage markers. Clin Cancer Res.

[30] Hoy SM (2018). Talazoparib: first global approval. Drugs.

[31] Eisenhauer EA, Therasse P, Bogaerts J, Schwartz LH, Sargent D, Ford R, Dancey J, Arbuck S, Gwyther S, Mooney M, Rubinstein L, Shankar L, Dodd L, Kaplan R, Lacombe D, Verweij J (2009). New response evaluation criteria in solid tumours: revised RECIST guideline (version 1.1). Eur J Cancer.

[32] Yu Y, Chung CH, Plotka A, Quinn K, Shi H, Papai Z, Nguyen L, Wang D (2019). A phase 1 mass balance study of (14) C-labeled talazoparib in patients with advanced solid tumors. J Clin Pharmacol.

[33] Ji J, Kinders RJ, Zhang Y, Rubinstein L, Kummar S, Parchment RE, Tomaszewski JE, Doroshow JH (2011). Modeling pharmacodynamic response to the poly(ADP-Ribose) polymerase inhibitor ABT-888 in human peripheral blood mononuclear cells. PLoS ONE.

[34] NCI-DCTD. https://dctd.cancer.gov/ResearchResources/biomarkers/PolyAdenosylRibose.htm

[35] Wang LH, Pfister TD, Parchment RE, Kummar S, Rubinstein L, Evrard YA, Gutierrez ME, Murgo AJ, Tomaszewski JE, Doroshow JH, Kinders RJ (2010). Monitoring drug-induced gammaH2AX as a pharmacodynamic biomarker in individual circulating tumor cells. Clin Cancer Res.

[36] Navas T, Kinders RJ, Lawrence SM, Ferry-Galow KV, Borgel S, Hollingshead MG, Srivastava AK, Alcoser SY, Makhlouf HR, Chuaqui R, Wilsker DF, Konate MM, Miller SB, Voth AR, Chen L, Vilimas T, Subramanian J, Rubinstein L, Kummar S, Chen AP, Bottaro DP, Doroshow JH, Parchment RE (2020). Clinical evolution of epithelial-mesenchymal transition in human carcinomas. Cancer Res.

[37] Yu Y, Durairaj C, Shi H, Wang DD (2020). Population pharmacokinetics of talazoparib in patients with advanced cancer. J Clin Pharmacol.

[38] Litton JK, Scoggins ME, Hess KR, Adrada BE, Murthy RK, Damodaran S, DeSnyder SM, Brewster AM, Barcenas CH, Valero V, Whitman GJ, Schwartz-Gomez J, Mittendorf EA, Thompson AM, Helgason T, Ibrahim N, Piwnica-Worms H, Moulder SL, Arun BK (2020). Neoadjuvant talazoparib for patients with operable breast cancer with a germline BRCA pathogenic variant. J Clin Oncol.

[39] NLM. https://www.ncbi.nlm.nih.gov/clinvar/variation/55480/?new_evidence=true

[40] Kim C, Wang XD, Yu Y (2020). PARP1 inhibitors trigger innate immunity via PARP1 trapping-induced DNA damage response. Elife.

[41] Redon CE, Nakamura AJ, Zhang YW, Ji JJ, Bonner WM, Kinders RJ, Parchment RE, Doroshow JH, Pommier Y (2010). Histone gammaH2AX and poly(ADP-ribose) as clinical pharmacodynamic biomarkers. Clin Cancer Res.

[42] O'Sullivan Coyne G, Karlovich C, Wilsker D, Voth AR, Parchment RE, Chen AP, Doroshow JH (2022). PARP inhibitor applicability: detailed assays for homologous recombination repair pathway components. Onco Targets Ther.

[43] Shibue T, Weinberg RA (2017). EMT, CSCs, and drug resistance: the mechanistic link and clinical implications. Nat Rev Clin Oncol.

[44] Dongre A, Weinberg RA (2019). New insights into the mechanisms of epithelial-mesenchymal transition and implications for cancer. Nat Rev Mol Cell Biol.

[45] Ordonez LD, Hay T, McEwen R, Polanska UM, Hughes A, Delpuech O, Cadogan E, Powell S, Dry J, Tornillo G, Silcock L, Leo E, O'Connor MJ, Clarke AR, Smalley MJ (2019). Rapid activation of epithelial-mesenchymal transition drives PARP inhibitor resistance in Brca2-mutant mammary tumours. Oncotarget.

[46] Lamouille S, Xu J, Derynck R (2014). Molecular mechanisms of epithelial-mesenchymal transition. Nat Rev Mol Cell Biol.

[47] Polyak K, Weinberg RA (2009). Transitions between epithelial and mesenchymal states: acquisition of malignant and stem cell traits. Nat Rev Cancer.

[48] Medema JP (2013). Cancer stem cells: the challenges ahead. Nat Cell Biol.

[49] Lonn P, van der Heide LP, Dahl M, Hellman U, Heldin CH, Moustakas A (2010). PARP-1 attenuates Smad-mediated transcription. Mol Cell.

[50] Trelford CB, Dagnino L, Di Guglielmo GM (2022). Transforming growth factor-beta in tumour development. Front Mol Biosci.

