# Supplementary material for: Pharmacodynamic effects of the PARP inhibitor talazoparib (MDV3800, BMN 673) in patients with BRCA-mutated advanced solid tumors
Source: Cancer Chemother Pharmacol. 2023 Nov 27;93(3):177–89. doi: 10.1007/s00280-023-04600-0 (PMC10902014; doi:10.1007/s00280-023-04600-0)
Supplement: Supplementary file 1 — Supplementary file1 (DOCX 1214 KB) [file 280_2023_4600_MOESM1_ESM.docx]

**Supplemental** **Table S1. Patient PD Summary**

| **Patient number, best response, cycles on study, and prior platinum** | **Diagnosis (biopsy site) and BRCA mutation** | **PAR levels from first pass biopsies:**  **pg/μg protein and % change** | **γH2AX levels from second pass biopsies: % Nuclear Area Positive** | **Epithelial-mesenchymal phenotype from second pass biopsies: Log(V/E) and *p*-value** |
| --- | --- | --- | --- | --- |
| 1  PD (2 cycles)  Prior Pt | Uterine Sarcoma (lung)  BRCA2 g3237delCA | Pre: 21.30  Post: 5.00  -77% | Pre: 1.0%  Post: 6.3% | Pre: 2.56  Post: 4.58  *p* < 0.001 |
| 2  PR (18 cycles)  Prior Pt | Ovarian (liver)  BRCA1 gR1751X (5379C>T) | Pre: 2.00  Post: 1.90  -5% | Pre: NA*  Post: NA* | Pre: NA*  Post: NA* |
| 3  PR (10 cycles)  Prior Pt | Ovarian (neck lymph node)  BRCA1 g187delAG | Pre: NA*  Post: 2.90 | Pre: NA*  Post: 6.2% | Pre: NA*  Post: not analyzed |
| 4  PR (8 cycles)  No prior Pt | Prostate (cervical node)  BRCA2 gK1872X (5842A>T) | Pre: 29.30  Post: 1.20  -96% | Pre: 1.8%  Post: 5.2% | Pre: -0.64  Post: -0.12  *p* < 0.001 |
| 5  PR (6 cycles)  No prior Pt | Prostate (liver)  BRCA1 gQ1756fs*74 | Pre: 27.90  Post:2.40  -91% | Pre: 0.3%  Post: 1.7% | Pre: -1.68  Post: -1.26  *p* < 0.001 |
| 6  SD (6 cycles)  Prior Pt | Breast (Supraclavicular  node)  BRCA1 g 2576delC | Pre: 6.90  Post: 0.80  -88% | Pre: 2.5%  Post: 4.7% | Pre: 0.41  Post: 0.28  *p* > 0.05 |
| 7  SD (8 cycles)  No prior Pt | Prostate (iliac bone)  BRCA2 g c.476-2A>G | Pre: NA*  Post: NA* | Pre: NA*  Post: NA* | Pre: NA*  Post: NA* |
| 8^  PR (8 cycles)  No prior Pt | Breast (liver)  BRCA2 g3917delC | Pre: 34.5  Progression^: 7.20  -79% | Pre: 0.5%  Progression^: 1.0% | Pre: -1.13  Progression^: -0.38  *p* < 0.001 |
| 9  PD (< 1 cycle)  Prior Pt | Pancreatic (omentum)  BRCA1 g3171ins5 | Pre: 17.61  Post: 0.34  -98% | Pre: 0.9%  Post: 2.6% | Pre: -1.42  Post: -1.24  *p* > 0.05 |

^Patient 8 post-dose biopsy was taken at progression (after cycle 8), not day 8 of treatment as for all other patients. *Biopsy tissue unanalyzable due to it containing less than 5% tumor cells or significant damage.

**Supplemental Figure S1. Patient *BRCA1/2* mutations**


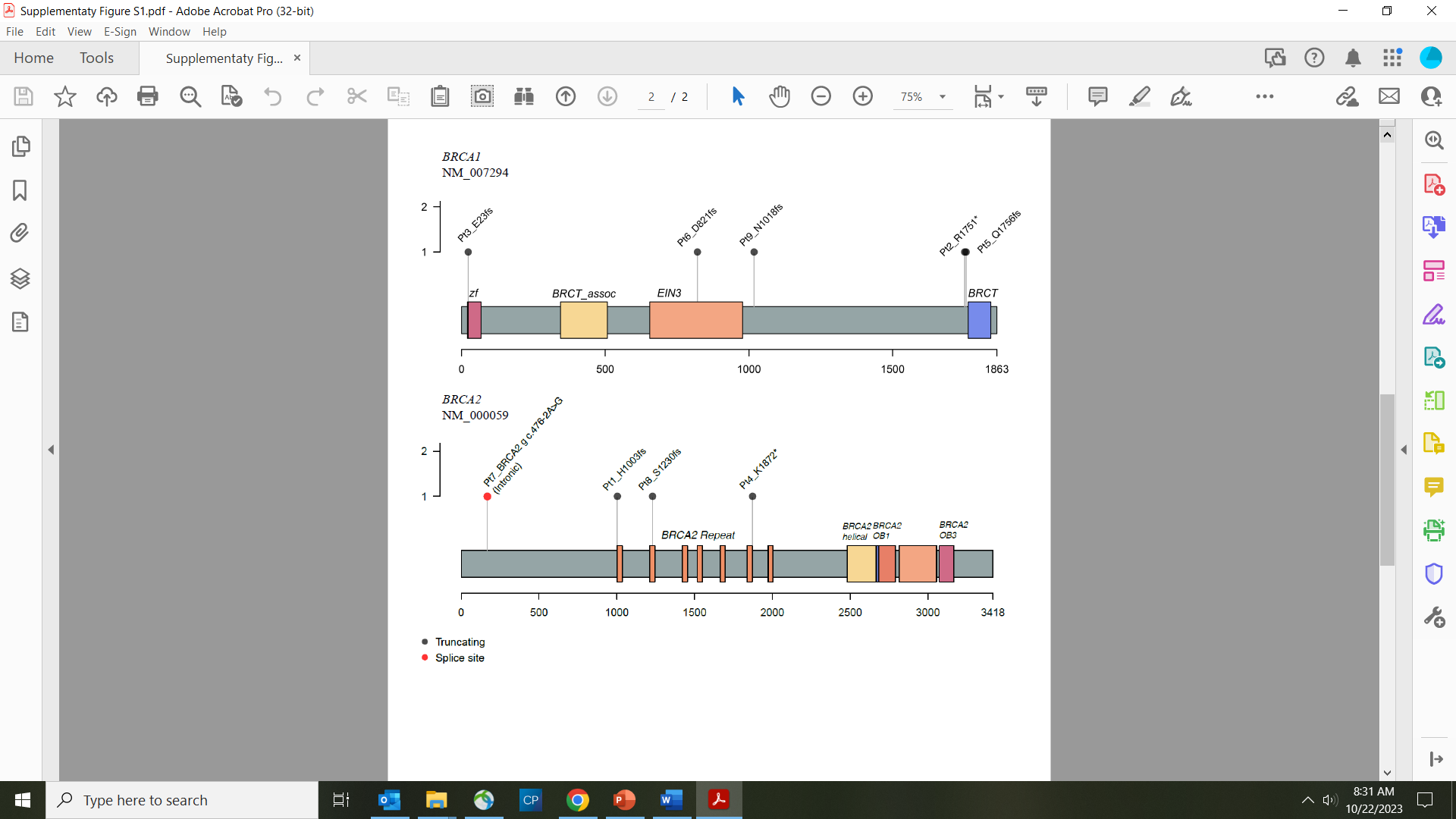


**Patient *BRCA1/2* mutations.** The distribution of eligibility mutations within the *BRCA1* and *BRCA2* genes for the 9 patients on trial (note that the *BRCA2* g c.476-2A>G mutation of patient #7 is intronic). *BRCA1* domains: zf, zinc finger C3-HC4; BRCT-assoc., serine-rich domain associated with BRCT; EIN-3, ethylene-insensitive 3; BRCT, BRCA1 C terminus domain. *BRCA2* domains: BRCA2-helical; BRCA2-OB1, oligonucleotide/ oligosaccharide binding domain 1; BRCA2-OB3, oligonucleotide/oligosaccharide-binding domain 3.


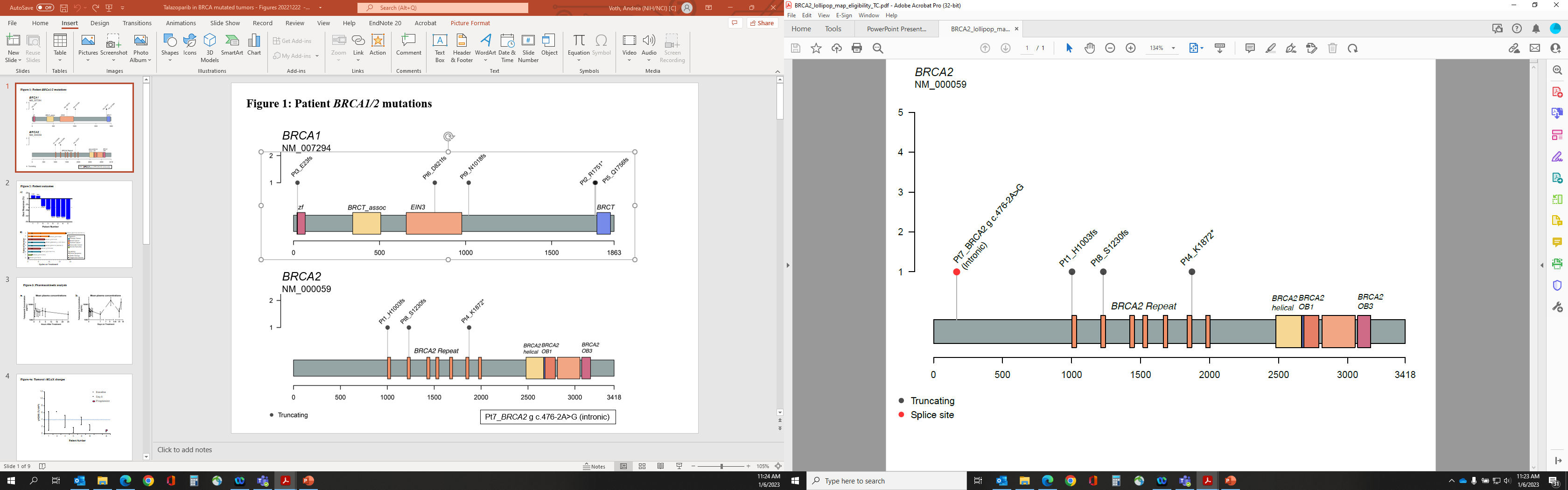


*BRCA2*

NM_000059
